# Abstracts and Gleanings

**Published:** 1877-01-20

**Authors:** 


### Abstracts and Gleanings.

*Epilepsy.*—Dr. A. McLane Hamilton, Physician to Epileptic Hospital, Blackwell's Island, in an interesting paper on Epilepsy in the *Chicago Medical Journal*, urges the neeessity for discovering the exciting cause in this obstinate and troublesome affection. He affirms that the causes are not so frequently idiopathic as we are want to suppose. The treatment has been too empirical and routine, and that greater care in seeking the causes, and detecting the varied and delicate shades in epilepsy, would lead to better results in treatment. Reflex causes are at the root of many cases—among them uterine and ovarian irritation are frequent causes, and in children the organs of digestion, etc.

In syphilitic epilepsy pain precedes the attack. It is generally curable.

The leading indications in treating epilepsy are—

1. Removal of exciting cause, if possible.
2. The diminution of exaggerated reflex susceptibility of the medulla.
3. Equalization of cranial circulation.
4. Abortion of paroxysms.
5. Improvement of general condition.

The remedies mentioned are the bromides, belladonna, digitalis, strychnine, ergot, arsenic, amyl nitrite, tri-nitro-glycerin, cod liver oil.

The bromides are frequently given to excess, producing prolonged bromism. He has had best results from sixty grains in twenty-four hours, and smaller quantities may suffice in some cases.

Belladonna is particularly adapted to cases where there are centers of irritation, as in gastric epilepsy, having the property of blunting reflex susceptibility.

Ergot controls the cranial circulation, and diminishes congestion at the floor of the first ventrical.

Arsenic is excellent for its anti-periodic and alterative action.

Digitalis is useful when there is irregularity of the heart's action, sluggish circulation, etc.

Amyl nitrite and tri-nitro-glycerin produce congestion of the brain, and are useful in cases where there is cerebral anæma.

The latter is as powerful a medicinal agent, as it is explosive. A tenth part of a drop touched to the tongue produces rapid cerebral hyperdæmia. An alcoholic solution is not explosive. One drop may be dissolved in ten drops of alcohol, and this again diluted, so that a dose of ten drops will contain one-tenth of a drop of the remedy.

*Post Partem Hemorrhage.*—Dr. Herndon, in the Virginia *Medical Monthly*, reports a case of post partem hemorrhage wherein, having failed with all the usual remedies, he succeeded by injecting dr. vj tincture of iodine into the uterus. The long nozzle of a syringe was introduced for this purpose, up to the fundus uteri. The contraction of the uterus was immediate and permanent, and the hemorrhage effectively suppressed.

*Atropia as an Antidote to Hydrocyanic Acid.* JACKSON. (*Druggists' Circular*, January, 1876.)—In experimenting on dogs, Dr. J. says: Sulphate of atropia, in doses of one-fourth of a grain to one grain, injected under the skin, gave prompt relief in every case, even when large doses of the acid had been given. When the two poisons are administered at the same time none of the effects of prussic acid are developed; but if as much as a grain of sulphate of atropia be injected, all the symptoms of atropia poisoning are observed. In some instances the antidote was withheld until the animal would fall down, and the respirations would be as few as six per minute, the dog being unconscious, then one-fourth grain of the antidote would relieve him immediately. J. S. K.

*Bromohydrate of Quinine.*—The bromohydrate of quinine contains more of the alkaloid than the sulphate, and yet produces less cinchonism, the result, doubtless, of the soothing or hypnotic properties of the bromine in combination. It is well adapted to nervous or irritable subjects, particularly to females suffering with dysmenorrhœa, neuralgia, or headache attended with cerebral hyperdœmea. The dose is five to fifteen grains in pill or hypodermically.

*Hip Joint Amputation.*—Two successful cases of amputation at the hip joint are reported in December number of the *N. Y. Medical Journal*, by Dr. Erskine Mason.

The circular method was adopted in both cases. Esmarch's elastic bandage was applied upon the limb at the point of section, and the abdominal tourniquet was applied to compress the aorta. They are the first instances of the use of Esmarch's bandage in this operation.

The tourniquet used was the one known as May's modification of Signoroin's. It was shown that the compressing pad in this instrument is too large. Hence, the one which bears the name of Lister is thought better. The compression of the pad in one case was followed by peritonitis, yet the case recovered.

When the great mortality which has been heretofore reported of this operation is considered, the favorable result must be largely due to the great saving of blood in the method here practiced.

*Hyposulphite of Soda.*—The rapidity with which new agents are introduced, experimented with, lauded for a brief space for wonderful power, and hastily thrown aside, is well illustrated in the case of the hyposulphite of soda, which a few years ago was thrust forward and greatly extolled, and is now scarcely heard of. And yet we are satisfied it should not be so, as we believe that it is a very valuable agent.

It is particularly useful in obstinate eruptive diseases. A solution of one drachm to the ounce of water, locally applied, constitutes a pleasant and most effectual remedy for scabies, relieving very obstinate cases in a few days.

The same solution is very efficacious as a topical application in diphtheria. In apthous ulcerations and other forms of sore mouth, it is admirable.

*Salicylate of Soda.*—This salt is regarded as a powerful febrifuge. It reduces the temperature in fevers with great certainty and uniformity. Acts finely in rheumatic fever, and though not proven to possess strong anti-periodic properties, will, in all probability, be found a powerful auxiliary to quinine in the treatment of malarial affections.

*Visical Disturbances from Diphtheria.*—Prof. Seely read a paper before the Cincinnati Academy of Medicine, in regard to disturbances of vision during convalescence from diphtheria.

"The phenomena, subjective and objective, usually are: inability to read fine print, fatigue of the eyes, double vision, vertigo and squint."

These symptoms are attributed to inflammatory action in the periphery of the optic nerve at its entrance into the eyeball, resulting from diphtheritic poison. In a case which was somewhat anæmic, repeated dry cupping to the temples, and the administration of the mur. tinc. of iron was followed by relief.

*Scarlatinal Albuminaria.*—Dr. Bell, in *Transactions of the Pennsylvania Medical Society*, says that in scarlatinal albuminaria he has found the following prescription superior to all other remedies:

R. Scoparius, (the tops,) oz.ss;
Water, ojss.

Boil down to one pint, and give one to two tablespoonsful every four to six hours.

*Petroleum in Cutaneous Affections.*—The following ointment has been found highly efficacious in cutaneous affections, particularly in porigo and other parasitic diseases of the scalp:

R. Petroleum,.................oz.iv;
Lard, (melted,).............oss;
Oil of lavender,............gtt.xv;

M. and stir at a gentle warmth until thoroughly blended.

It is destructive to vermin, and is well adapted to the cure of mange of dogs, swine or other animals. In the human subject it may be applied twice a day, the part being first cleansed with castile soap and warm water.

*Salicylate of Soda as an Antipyretic.*—Dr. Nathan (*Deut. Zeit. fur Prakt. Med.—London Medical Record*, May 15, 1876) has used this remedy in twelve cases, viz: nine of enteric fever, one of phthisis, one of osteomyelitis of both tibiæ, and one of subperiosteal resection of the femur. In all cases the temperature, which was excessively high, was reduced by salicylate of soda. The best antipyretic action was obtained by large doses often repeated—two drachms, repeated in two hours until an effect was produced. After the temperature had become normal, smaller doses prevented the further rise of temperature. The remedy is given in water, one part to four or five. As an antipyretic he regards the salicylate of soda as preferable to quinine, digitalis, and veratria.—*Detroit Rev. Med.*

Acute Rheumatism Treated by Salicin.—Dr. T. McLagan (*London Lancet*) reports eight carefully observed cases of rheumatism treated by salicin. From these he concludes:

1. We have in salicin a valuable remedy in the treatment of acute rheumatism.

2. The more acute the case, the more marked the benefit produced.

3. In acute cases, its beneficial action is generally apparent within twenty-four—always within forty-eight—hours of its administration in sufficient dose.

4. Given thus at the commencement of the attack, it seems sometimes to arrest the course of the malady as effectively as quinine cures an ague, or ipecacuanha a dysentery.

5. The relief of pain is always one of the earliest effects produced.

6. In acute cases, relief of pain and a full temperature generally occur simultaneously.

7. In sub-acute cases, the pain is sometimes decidedly relieved before the temperature begins to fall. This is especially the case when, as is frequently observed in those of nervous temperament, the pain is proportionally greater than the abnormal rise of temperature

8. In chronic rheumatism, salicin sometimes does good where other remedies fail, but it also sometimes fails where others do good.—*Detroit Rev. Med.*

*Chronic Dysentery—Cured by Topical Treatment.*—Dr. T. G. Thomas (*New York Medical Journal*, January 1876) reports a case of chronic dysentery of five years' standing cured by three applications of nitric acid to the ulcerated rectum. The patient was anæsthetized—placed in the left lateral position, and after stretching the sphincter ani by the finger, a long duck-bill speculum was introduced. This was held by the nurse, exactly as in vaginal examinations, while by a depressor the anterior rectal wall was pressed downwards. The whole rectal canal was now exposed, to the sigmoid flexure. By syringe it was cleansed of all fæcal matters. Throughout its whole extent the exposed intestine was seen swollen, œdematous, hanging in hæmorrhoidal masses studded with deep ulcers, having grayish bottoms. It was greatly engorged and presented that deep red, almost violet hue, which is seen in the throat in cases of diphtheria. By cotton on a rod, nitric acid was lightly applied to the ulcers and swollen mucous membrane. After the third application, within a period of one month the patient was entirely well.—*Detroit Rev. Med.*

*Torsion—Its Advantages over Ligature in Arresting Hemorrhage.*—M. Tillaux, in a lecture at a Paris hospital (*Brit.

*Med. Jour.*, May 20, 1876) stated that he had for five years used torsion for all arteries, large or small. He believed that torsion is applicable to all arteries, especially the larger ones. A single pair of forceps is sufficient, and not two pairs, as employed in England and elsewhere. The artery should be seized obliquely and not longitudinally, and in such a manner that the three coats, in their entire breadth, should be included in their grip. The torsion, or twisting, should then be practiced until the portion seized becomes detached. Torsion is applicable to atheromatous, or inflamed arteries, as well as arteries in a healthy condition.

Torsion favors union by the first intention, owing to the absence of a foreign body, as in the case of ligatures. Like ligatures, torsion prevents primary hemorrhage; but it acts more effectually in preventing secondary hemorrhage. M. Tillaux asserts that, although he has employed torsion in about a hundred cases of capital operations, he has never had a single case of primary or secondary hemorrhage.

One of the attending surgeons to the Philadelphia hospital has for many years used torsion exclusively, with, on the whole, excellent success. He has devised an ingenious apparatus for twisting arteries.—*Detroit Med. Rev.*

*Lister's Autiseptic Method in Ovariotomy.*—Lister's antiseptic method in which carbolic spray and carbolized ligatures, etc., are used in operations, is attracting much attention in certain of the Northern hospitals. Dr. J. Marion Sims, N. Y., reports a case of ovariotomy successfully treated by this method. Dr. Sass was invited to use his apparatus for the application of the carbolic spray. The following account of the operation we find in the *Medical Record*, of New York:

The operation was done on Thursday, the 23d November last. I am particular in fixing the date, because I believe it inaugurates a new departure in ovariotomy.

Dr. Sass directed the spray, which covered the seat of operation with a delicate carbolic mist. The hands, sponges, and instruments were all dipped in carbolic water. The operation and dressing lasted forty minutes, the spray being kept up all the time. It could have been continued two hours, if necessary. There were no adhesions. The peritoneal cavity contained six or eight ounces of a reddish serum. The peritoneal membrane was everywhere deeply congested. This fact explains the presence of reddish serum, and the previous attack of peritonitis.

The pedicle was very short, and at least three inches broad. It was tied in three sections with strong twine, and drawn out and fixed in the lower angle of the wound, clamp-fashion.

The external incision was closed by sutures, and a carbolized dressing applied.

The pulse never rose above 90, nor the temperature over 101.

Convalescence was fully assured in forty-eight hours, and the patient is now quite well. The tumor was polycystic, on right side, and weighed fifteen pounds.

I hasten to lay this case before the profession merely to urge the adption of Lister's antiseptic method in ovariotomy, which, I am sure, will prove as valuable in this operation as it has in general surgery.

Dr. Sass's apparatus answered its purpose admirably, and I think he has rendered us a great service in bringing it before the profession at this time.

*India-Rubber Solution in Psoriasis.*—In the *London Lancet* we find a case of Psoriasis treated with india-rubber solution as follows:

A young woman, nineteen years of age, was admitted to the hospital on March 8th. She was of strumous appearance, but otherwise in fair health. Her affection had existed from childhood with varying degree of intensity, sometimes leaving her almost free, but with ever-recurring exascerbations. On admission her condition was as follows: Irregular patches, averaging about an inch and a half in diameter, were scattered over the body, and more thickly on the outer aspect of the limbs, and especially about the elbows and knees, extending over the wrists and hands. These patches had a base of inflamed and thickened cutis, overlying and firmly adherent to which were hard and dry crusts, the results of hyperplastic action of cuticular elements of the usual psoriasis type, in places exceeding the eighth of an inch in thickness, most abundantly developed around the elbows and knees. These crusts having been removed, to one side of the body and corresponding limbs the india-rubber solution was applied, while, to gain a comparative result, the other side was treated with mercurial and tar compounds. The affected places, on the side to which the india-rubber solution was used, at the end of three weeks became mere red markings, having lost in great measure their thickened bases, with but little tendency to the formation of scales remaining, while on the opposite side they continued to be produced, though with lessened activity, the inflammatory condition persisting. The india-rubber solution was then applied to the whole of the patches. She was discharged on May 3rd with only red stains to mark the former sites of the disease. The usual constitutional treatment was pursued.

In many of my other cases also one side or limb has in like manner been treated with these impermeable coverings, the corresponding parts receiving the more ordinary applications to allow of comparison of their actions.

The same local treatment is applicable also to some conditions of chronic eczema, but I have not as yet brought my experiments in that direction to a conclusion.

The solution is thus prepared:

R—India-Rubber.. ...............oz. ss.
Chloroform...... .. .....oz. xiss.

It is not adapted to acute cases.

*Dropsical Effusions.*—Dr. Chas. Burr, of carbondale, Pa., tells us in the Pennsylvania State *Transactions*, that he used to feel quite uncertain when called to a case of dropsy, but now he "can smile and promise a speedy cure." The reason of this change is his adopting in all such cases the following prescription;

R. Infus. digitalis......f.oz.iv.
Potassæ acetatis......oz.ss. M.

Dose—For an adult a tablespoonful, for a child a teaspoonful, every two hours.

If this prescription will exercise generally so happy an effect on physician and patient, our readers will thank us for reproducing it.—*Med. and Surg. Rep.*

We have used the cremor tartar with digitalis with similar results. W.

*Insect Destroyer.*—Take of quick lime four parts, sulphur one part. Break the lime into small pieces, mix the sulphur with it in an iron vessel, close as soon as the water is poured on.

This is a good whitewash for orchard trees, and is a preventive to mildew, blight, and all species of insects.

*Nasal Catarrh.*—The following prescription we extract from a newspaper, as having cured five obstinate cases of nasal catarrh :

1st R. Sulphate magnesia..........oz. iij
Port wine.......................Oj

M. S.—Take one teaspoonful before breakfast, as a blood purifier,

2d R. Tanic acid................ gr. xv
Glycerin,
Aquæ distil...............aa oz. j

M. S.—Sniffle a teaspoonful up the nostrils and into the throat frequently persevering until a cure follows.

*Exophthalmic Goitre*—The following case of this singular affection we extract from the *London Lancet*:

Case 1.—B. S——, aged twenty-two, a domestic servant, was admitted as an in-patient at the Stafford General Infirmary on Jan. 28th, 1876.

*Family history.*—Father and mother both alive, and enjoying good health. She has several brothers and sisters, and they are, and always have been, healthy. No evidence of any hereditary complaint whatever.

*Previous history.*—In the month of May last year, whilst feeling in every way in her usual health, she noticed that her eyes appeared more prominent than usual. This prominence kept gradually increasing, and a month or two afterwards she discovered a swelling on the right side of the neck, midway between the jaw and the collar bone. About the time she observed the swelling in the neck she began to be troubled with palpitations and shortness of breath; her menstration also became both scanty and irregular; her appetite failed; she lost both flesh and strength, becoming tired and exhausted by the least exertion. All her symptoms gradually increased in severity, and she became an in-patient at the infirmary on the date already mentioned.

*Condition on admission.*—The first thing that attracted attention was the strange and enormous prominence of the eyes; these appeared as if being fairly pushed out of their sockets. The next feature observed was a very considerable swelling of the thyroid gland, the swelling being much greater on the right than on the left side of the neck, extending indeed from the right side right across the trachea to the left side, where it was not so large. . . . She complained of no pain, said her bowels were regular, but that she had not menstrated for some months previously. Temperature perfectly natural; urine natural; appetite uncertain. She said she had lost much flesh, and that she was easily fatigued. She did not admit that she had suffered from any emotional excitement either before her illness, or since it commenced. Her eyesight was in no way affected; objects either near or distant were seen with great clearness. An ophthalmoscopic examination did not discover any unnatural condition; the disc was particularly clear and distinct.

*Treatment.*—As she had an anæmic appearance, she had given to her small doses of iron, and with the view of modifying and regulating the heart's action, tolerable large doses of digitalis were administered three times a day. The bowels, which were rather prone to be constipated, were kept regular with the aid of medicine. She was allowed a liberal diet, and, whenever the weather would admit of it, she took exercise in the open air.

During the time she was in the in firmary her general health improved considerably, but the prominent symptoms were but little if any relieved.

*The Internal Administration of Chloroform.*—M. Jaillard, a French army *pharmacien*, after alluding to the difficulties that occur in the internal administration of chloroform, states that the following simple procedure is by far the best: The prescribed quantity of chloroform should be poured into from 100 to 120 grammes of milk (which may be either pure, or sweetened and aromatized with a few drops of cherry-laurel water), and then briskly stirred. The chloroform, in this way, becomes easily divided into an infinity of minute globules, exactly resembling in appearance the fat globules which exist in the milk, in the midst of which they remain suspended for an indefinite period.—*Med. and Surg. Rep.*

*Poisoning by Œnanthe Crocata.*—Dr. Foss relates nine cases of poisoning in children by this plant. The root was the portion eaten by the victims. It is said to have a sweet taste, which is soon followed by a sharp, pungent sensation. The plant is called the five-fingered root, and is used very frequently in some parts as a dressing in severe cases of whitlow. The symptoms of the poisoning were similar in all the cases. Fifteen minutes after eating the root they all became unconscious, and had convulsions at intervals of half a minute between each. The pupils of the eye were widely dilated, the eyes were fixed and never moved, and the retinæ were insensible to light. There was no paralysis, reflex excitability, or any tendency to vomiting or purging. The pulse, at the beginning of the attack in each case was about 100, but for several hours before death were imperceptible. Post mortem examination showed the left side of the heart to be filled with clotted blood, and the right side to be empty. Both lungs were congested, and upon section dark blood and frothy corruption oozed out. The blood-vessels of the membranes of the brain were congested with black blood, and so the substance of the cerebrum and cerebellum.—*The Practitioner*, October, 1876.

*Simple Method of extracting Foreign Bodies from the Œsophagus.*—Dr. Edmond Le Bele proposes the following means, by which, on two occasions, in the same individual, he extracted large bones from the œsophagus: A piece of iron wire, of medium thickness and about two feet in length, is bent on itself in the middle so as to form a small loop, in the form of a crochet, the size and shape of which will correspond to the form and volume of the foreign body; the extremity, which rests in the hand of the operator, is likewise bent in such a manner that traction can be exerted; finally, the whole wire is bent so as to correspond to the bucco-pharyngeal curve. The patient being placed in a convenient position, the head is fixed by the left hand of the surgeon, while the right introduces the wire into the œsophagus, keeping it along the posterior wall until it reaches the foreign body, when the movement of deglutition by the patient will often suffice to place the crochet on the inferior surface of the foreign body. As the metallic wire does not take up much room in the patient's throat, it impedes the breathing less than the bulky instruments usually employed, which may also injure the epiglottis.—*Rev. de Therap.—Bull. de la Soc. de la Sarthe.*

*Warm Water Injections in Dysentery.*—Dr. J. J. Reed, in *New York Medical Journal,* claims fine success in dysentery by the use of warm water injections of the temperature of 100° to 110°, as follows: "The method of administration is quite simple, and does not require the services of a skilled nurse, or expensive apparatus.

"The hips of the patient are slightly raised, by means of a pillow, and a basin of water of the requisite temperature is placed in the bed so as to allow the nates to rest on the edge of the vessel. The vaginal nozzle of a Davidson's syringe is then introduced into the rectum, and alongside of it the rectal smaller nozzle. A current of water is then kept up for ten minutes, the water passing through the vaginal nozzle into the rectum, and returning by a steady stream through the smaller one into the basin, without causing any inconvenience to the patient. If the disease is extensive, and the colon involved for a considerable distance, a long rectal pipe may be employed instead of the vaginal nozzle.

"The immediate effect on the patient is one of comfort, which lasts for about an hour.

"The injections are to be continued every two hours, till the active stage of the disease is past."

*Bromide of Potassium as a Caustic.*—M. Peyrand, of Libourne, has demonstrated that bromide of potassium is a caustic. "His first clinical experiment on the subject took place in April, 1874, when, by means of daily applications of powdered bromide, he effected the removal within twenty-eight days of an epitheliomatous growth on the face. He has since had equally good results from this treatment of atonic ulcers of the legs, rapid cicatrisation following the separation of sloughs produced by the application. In such cases he uses either the powder or an ointment of one part in five, or a mixture (one in ten) of glycerine and the bromide. In many skin affections, as chronic eczema, pityriasis, and acne, in phagedæna, ulcerative stomatitis, and many other local inflammatory disorders, he has found it of use. As a local hæmostatic, a solution of one in fifty has served for epistaxis, and as a general hæmostatic its success in many cases of hæmoptysis and metrorrhagia was very marked, where ergot, perchloride of iron, and rhatany had failed."—*London Lancet.*

*Concentrated Solution of Salicylic Acid.*—Perhaps the best formula yet offered for a concentrated solution of salicylic acid, miscible without precipitation with aqueous solutions, is the following, proposed by C. L. Mitchell (*American Jour Phar.*):

Salicylic acid, pure.........dr.ij.
Borax ...........................dr.j
Glycerine ..................... q. s.

Mix the borax and acid with half a fluid ounce of glycerine, and heat gently until dissolved, then add glycerine to make up one fluid ounce. This solution can be diluted with water to any extent without immediate precipitation, but if the volume of water reaches more than ten times that of the glycerine the mixture becomes cloudy after a time.

According to Chas. Baker (*American Journal af Pharmacy,* July, 1876) salicylic acid, when heated with twice its weight of olive oil, forms a homogeneous mixture, admirably adapted for application to surfaces. The oil separates to some extent on standing, but can be readily recombined by shaking.—*Detroit Rev. Med.*

*Jaborandi as a Galactagogue.*—Jaborandi, in doses of five grains of the powder, infused, and taken three times a day, has had a very decided effect in increasing the secretion of milk in several cases where mothers have had scarcely any flow, and have, in consequence, been unable to suckle their children. After taking the above-mentioned powders the flow has very soon been much more satisfactory, to the surprise of the patient, and somewhat to that of the doctor, as I have not hitherto had much faith in galactagogues. It is, however, necessary, if the breasts be not well developed, and therefore do not readily take to their secreting function, to continue the drug for some time, as the secretion will probably again fall off if the powders are discontinued too early. The resulting milk, I need scarcely say, agrees quite well with the infant, the jaborandi having no unfavorable effect upon its quality. The drug itself is said to have no such effect upon children as upon adults. The above property was indicated and the earliest accounts of the effects of jaborandi published by Dr. Ringer and others, by whom it was noticed that it increased the secretions of the skin and of the salivary and mammary glands.—*Brit. Med. Jour.*

*Treatment of Orchitis.*—For the treatment of this disease, Dr. L. D. Waterman, of Indiana, claims excellent results, in the *Practitioner*, with the following:

R. Tincturæ iodinii,
Aquæ ammoniæ,
Tincture opii,
Olei olivæ.

The iodine and ammonia are added in quantity just sufficient to be bearable, and only cause half-blistering of the skin, or exfoliation with a stinging sensation for a short time after application. Thus graduated to the supposed endurability, the free application of it is made to the entire surface of the scrotum, and the woolen cloth saturated with the liniment, with which it is hourly (if possible) applied, is wrapped around the scrotum, and left there continually. The pain ceases sometimes in three hours, always within twenty-four, and the effusion is correspondingly rapidly absorbed without tapping.—*Med. and Surg. Rep.*

*Method of Bandaging the Breast.*—Dr. L. A. Dugas (*Louisville Med. Jour.*, April, 1876) says that in treating mammitis or mammary abscess he uses the following method of bandaging: A bit of cotton or linen shirting, about ten inches wide, and long enough, is placed about the thorax and secured in front by digitations similar to other "many-tailed" bandages. This is to be applied from the axilla down, passing around the chest and over the mamma, and to be tied in front of the sternum. It effectually compresses both organs, and may be removed, loosened or tightened according to the exigencies of the case, without any difficulty whatever. If only one breast is affected, the bandage may be so split as not to cover the whole of the other. The child may be nursed through an aperture made in the bandage for the nipple. As soon as the congestion becomes painful or threatening, the bandage should be applied with such moderate tightness as will relieve pain, and it should be continued until the trouble has entirely subsided.

*Diseases of the Nose treated by Medicated Bougies.* Catti. (*Memorabilien*, 1876, No. 9.)—At a meeting of the Vienna Medical Society, Catti showed some gelatine bougies charged with astringent medicines—as sulphate of copper, zinc, carbolic acid, tincture of rhatany. They are from eight to eleven centimeters long, and from four to six centimeters thick. By a slow rotatory motion they are introduced into the nasal cavity, and by giving them a horizontal or slanting or perpendicular direction they can be inserted into the lower or middle nasal passage. The bougie inserted, the nostril is plugged with charpie or cotton, and in a few hours it will be entirely melted. By a regular continued use of such bougies Catti obtained very good results in the treatment of chronic catarrh of the nose and nasopharngeal space, and of the ozæna.—*Chicago Med. Journal.*

*Solution of Phosphorous in Glycerine.*—Phosphorous dissolves in alcohol only after prolonged heating, but it is rapidly taken up when agitated with warm glycerine, and the solution may then be diluted with warm alcohol without separation of the phosphorous.—*Detroit Rev. Med.*

*Mucilage of Acacia*, if prepared with tolu water, instead of simple water, will keep for several months. The tolu water is made by triturating fl. dr. ij tinct, tolu with dr. iv carb. magnesium, then with Oij. of water, and filtering.—*Detroit Rev. Med.*

*Boldo.*—Boldo is a South American plant, a native of Chili. Its leaves, bark and blossoms possesses aromatic odor resembling turpentine and camphor. An essential oil has been obtained from the leaves, and an alkaloid called "boldine." It has the property of stimulating the liver and aiding digestion. One gramme of the tincture may be given at a dose, three times daily.

*Absorption of Iodine by the Skin.*—In a paper in *L' Union Med.*, it is shown that iodine poisoning resulted from the free application of iodine to the scalp of a child, in the treatment of ring-worm. It is not proper, therefore, to apply the remedy on extensive surfaces in children. The iodism was attended with albuminaria of a marked character.

*Quinine in India.*—" In the treatment of malarious cases in India, it is found that quinine loses its effect after a short time. Liquor arsenicalis in chronic cases, and the nitric acid bath for children, are the remedies now in favor in that country. The nitric acid is used much in the manner of the nitro-muriatic bath of the older writers."

Dr. Grave, in *Jour. of Mat. Med.*, 1868, reports seventy-six out of seventy-eight cases of intermittent fever, promptly cured by the following:

| | | |
|---|---|---|
| R. | Soda hyposulphite, | grs. iv |
| | Syrupus simplex, | dr. j |
| | Aquæ, | oz. ij |

M. Dose—Half tablespoonful every two hours.

*Coffee Leaf.*—It is said that the leaf of the coffee tree makes a beverage superior to the coffee, as usually prepared, and that it is in common use in Australia. Let it be introduced into Florida.

